# Sex-Specific Associations of Risks and Cardiac Structure and Function With Microalbumin/Creatinine Ratio in Diastolic Heart Failure

**DOI:** 10.3389/fcvm.2020.579400

**Published:** 2020-10-07

**Authors:** Fang-Fei Wei, Ruicong Xue, Yuzhong Wu, Weihao Liang, Xin He, Yuanyuan Zhou, Marvin Owusu-Agyeman, Zexuan Wu, Wengen Zhu, Jiangui He, Jan A. Staessen, Yugang Dong, Chen Liu

**Affiliations:** ^1^Department of Cardiology, The First Affiliated Hospital of Sun Yat-sen University, Guangzhou, China; ^2^NHC Key Laboratory of Assisted Circulation, Sun Yat-sen University, Guangzhou, China; ^3^Studies Coordinating Centre, Research Unit Hypertension and Cardiovascular Epidemiology, Department of Cardiovascular Sciences, University of Leuven, Leuven, Belgium; ^4^Non-profit Research Institute (NPA) Alliance for the Promotion of Preventive Medicine, Mechelen, Belgium; ^5^National Guangdong Joint Engineering Laboratory for Diagnosis and Treatment of Vascular Disease, Guangzhou, China

**Keywords:** heart failiure, chronic kindney disease, echocardiography, microalbuminuria, risk stratification, gender

## Abstract

**Background:** Heart failure with preserved ejection fraction (HFpEF) affects women more frequently than men. However, data on sex-specific associations of adverse health outcomes and left ventricular structure and function and with microalbuminuria in patients with HFpEF are scarce.

**Methods:** In 1,334 participants enrolled in the Treatment of Preserved Cardiac Function Heart Failure with an Aldosterone Antagonist (TOPCAT) Trial, we estimated the sex-specific multivariable-adjusted risk and LV traits with urine microalbumin/creatine ratio (ACR), using Cox or linear regression.

**Results:** In total, 604 (45.3%) were women. In multivariable-adjusted analyses, a doubling of ACR in both men and women was associated with higher posterior (+0.014 cm, *p* = 0.012/+0.012 cm, *p* = 0.033) wall thickness and left ventricular mass index (+2.55 mg/m^2^, *p* = 0.004/+2.45 mg/m^2^, *p* = 0.009), whereas was also associated with higher septal (+0.018 cm, *p* = 0.002) and left atrial volume index (+1.44 mL/m^2^, *p* = 0.001) in men. ACR was a key predictor of all-cause (HR, 1.11; *p* = 0.006) and cardiovascular (HR, 1.17; *p* = 0.002) death in women, whereas in men ACR was associated with HF hospitalization (HR, 1.23; *p* < 0.001), any hospitalization (HR, 1.06; *p* = 0.006), and myocardial infarction (HR, 1.19; *p* = 0.017). The interactions of sex with ACR were significant for hospitalization for heart failure and any hospitalization (*p* ≤ 0.034).

**Conclusions:** Outcomes and cardiac structure and function in patients with HFpEF appear to be influenced by ACR that vary according to sex. In men, ACR was significant associated with LV diastolic function, hospitalization, and myocardial infarction, whereas in women was associated with mortality.

## Introduction

Heart failure (HF) is a major global health problem (1) and the leading cause of morbidity and mortality worldwide (2). HF with preserved ejection fraction (HFpEF) accounts for ≈50% of cases (3). HFpEF is characterized by multiple co-morbidities, including chronic kidney disease (CKD) (4). The co-existence of HF and CKD is associated with extremely poor prognosis (5–7). The urine albumin/creatine ratio (ACR) is commonly used to evaluate the severity of CKD (8). In the general population (9), or in the patients with hypertension (10), diabetes mellitus (11), and heart failure (5), albuminuria behaves as a well-established risk factor for mortality.

Sex is a critical determinant of cardiovascular structure and function and various adverse health outcomes. Women are more likely to develop HFpEF than men (12, 13). However, our literature review did not reveal any previous study that addressed the possible sex differences in the cardiac structure and function and adverse health outcomes in relation to urine microalbumin/creatinine ratio in the setting of HFpEF. Thus, we addressed this knowledge gap to explore the sex-specific associations of cardiac structure and function and adverse health outcomes with urine ACR using data of the TOPCAT (the Treatment Of Preserved Cardiac Function Heart Failure with an Aldosterone Antagonist Trial; NCT00094302) (14, 15).

## Methods

### Study Participants

The TOPCAT study was an international, multicenter, randomized, double-blind, placebo-controlled trial. The TOPCAT trial was designed to investigate whether spironolactone improved clinical outcomes in patients with HFpEF compared with placebo. The TOPCAT study complies with the Declaration of Helsinki and the Institutional Review Board has approved this research. There were 3,445 participants at 233 sites in six countries randomly assigned to spironolactone or placebo. Inclusion criteria were age ≥50 years, ≥1 sign and at least one symptom of HF, left ventricular (LV) ejection fraction ≥45%, controlled systolic blood pressure, and serum potassium <5 mmol/L. All patients signed informed consent prior to randomization. A detailed description of the TOPCAT design and protocol has previously been reported (14). To obtain access to the TOPCAT data, we first registered at the website of the Biologic Specimen and Data Repository Information Coordinating Center of National Heart, Lung, and Blood Institute (NHLBI; https://biolincc.nhlbi.nih.gov/). Next, we submitted a request for accessing the TOPCAT data along with a protocol for the intended *post-hoc* analysis and the approval by the ethics committee of the First Affiliated Hospital, Sun Yat-sen University, Guangzhou, China. After we signed a Research Materials Distribution Agreement, NHLBI transferred anonymized data. Of 3,445 patients, 1,334 patients had qualified urine ACR measurement.

### Laboratory Measurement

The participants were asked to provide a spot urine specimen to measure urine ACR at baseline. Laboratory measurements for urine chemistries were performed locally at the enrolling site. Albuminuria was defined by urine ACR ≥30 mg/g (16).

### Echocardiographic Measurement

Echocardiographic measurements and laboratory measurement for urine ACR were performed at the baseline visit. Of 3,445 randomized HFpEF patients, 935 (27.1%) underwent echocardiography prior to the initiation of randomized treatment (17). The echocardiographic examination was performed according to the recommendations of the American Society of Echocardiography, as preciously described (17). Quantitative measurements on all study echocardiograms were performed by dedicated analysts at the core laboratory blinded to clinical information. Previous publications describe the procedures applied for acquisition and the off-line analysis of the echocardiographic measurements in detail (14). In this study, we statistically analyzed LV structure including LV dimensions, wall thickness and mass index, diastolic function including left atrial volume index, transmitral blood flow, and mitral annular tissue velocities, and systolic function including ejection fraction and longitudinal strain. Intra-observer variability for key echocardiographic measures of cardiac structure and function have been previously reported (15, 18). Intra-observer variability performed in 60 studies, was as follows: wall thickness: coefficient of variation 12%, bias 0.02 ± 0.1 cm; LV end-diastolic volume: coefficient of variation 12%, bias 1.6 ± 10.5 mL; LV end-systolic volume: coefficient of variation 18%, bias 2.6 ± 5.9 mL; LV EF: coefficient of variation 6.6%, bias 2.0 ± 4.3%; tissue Doppler imaging e': coefficient of variation 7.0%, bias 0.1 ± 0.4 cm/s; E/e' ratio: coefficient of variation 11%, bias 0.2 ± 1.2 (15, 18).

### Ascertainment of Endpoints

The primary outcome was a composite of all-cause death, non-fatal myocardial infarction, non-fatal stroke, or hospitalization for HF. In the present study, we also included all-cause death, cardiovascular death, HF hospitalization, any hospitalization, myocardial infarction, and stroke. All events were adjudicated by a clinical end-point committee at Brigham and Women's Hospital, Boston, USA, according to pre-specified criteria (14). More detailed information on the evaluation of outcomes has previously been reported (14, 15).

### Statistical Analysis

For database management and statistical analysis, we used SAS software, version 9.4 (SAS Institute Inc., Cary, NC), maintenance level 5. We compared means and proportions by the large-sample *z*-test or ANOVA and Fisher exact test, respectively. We normalized the distributions of urine ACR by a logarithmic transformation. The central tendency (spread) was represented by the arithmetic mean (SD) for normally distributed variables and by the geometric mean (interquartile range) of logarithmically transformed variables. A *p*-value of ≤ 0.05 was considered statistically significant.

In exploratory analyses, we determined differences in participants' baseline characteristics across thirds of the urine ACR. In unadjusted and multivariable-adjusted linear regression analyses, we expressed association sizes of the echocardiographic indexes for a doubling of a urine ACR. In multivariable-adjusted analyses, in line with previous publications (5), we accounted for randomly assigned treatment (spironolactone vs. placebo), sex, age, ethnicity, body mass index, systolic blood pressure, heart rate, current smoking, use of medications (diuretics, β blockers, angiotensin converting enzyme inhibitors, angiotensin receptor blockers, calcium-channel blockers, lipid-lowering drugs, aspirin, other cardiovascular medications, and hypoglycemic agents), and prevalence of diabetes mellitus and dyslipidemia. We applied Cox proportional hazards regression models to assess the associations of the urinary ACR with the primary endpoint, all-cause mortality, cardiovascular mortality, hospitalization, myocardial infarction, and stroke. We also compared the sex differences in the associations of urine ACR with various adverse health outcomes. In sensitivity analyses, concentrations of serum creatinine were added simultaneously to the model with urine ACR to assess risk associated with increased ACR, independent of renal dysfunction. Sensitivity analyses excluding those on spironolactone treatment were performed to assess the association between various adverse health outcomes and urine ACR.

## Results

### Baseline Characteristics of Participants

Among 1,334 participant, 604 (45.3%) were women. In all participants, mean values (±SD) were 70.9 ± 9.6 years for age, 33.0 ± 7.6 kg/m^2^ for body mass index, and 127.9 ± 15.1/73.2 ± 11.6 mm Hg for systolic/diastolic blood pressure. Table 1 lists the characteristics of patients by tertiles of the urine ACR. Across increasing categories (Table 1), systolic blood pressure, serum creatinine, the prevalence of diabetes mellitus and dyslipidemia, the frequencies of use of β-blockers, calcium channel blockers, hypoglycemic agents increased (*p* ≤ 0.005).

**Table 1:** Baseline characteristics of participants by tertiles of the microalbumin/creatinine ratio distribution.

**Characteristics**	**Category of microalbumin/creatinine ratio**			***p*-value**
Limits, mg/g	< 14.0	14.0–61.9	≥61.9	
Number (%) with characteristic	445 (33.4)	441 (33.1)	448 (33.6)	0.97
Women	208 (46.7)	196 (44.4)	200 (44.6)	0.75
**Race**				
White	377 (84.7)	372 (84.4)	353 (78.8)^*^	0.032
Black	56 (12.6)	44 (10.0)	75 (16.7)^†^	0.011
Asian	2 (0.45)	5 (1.13)	6 (1.34)	0.37
Others	12 (2.7)	20 (4.5)	18 (4.0)	0.33
Current smoking	36 (8.1)	36 (8.2)	32 (7.1)	0.82
Office hypertension	389 (87.4)	398 (90.2)	402 (89.7)	0.35
Diabetes mellitus	159 (35.7)	162 (36.7)	219 (48.9)^‡^	<0.001
Dyslipidemia	272 (61.1)	276 (62.6)	332 (74.1)^‡^	<0.001
eGFR <60 mL/min/1.73 m^2^	176 (39.6)	204 (46.3)^*^	224 (50.0)	0.006
**Medications**				
β-blockers	335 (75.4)	329 (74.6)	371 (82.8)^†^	0.005
Diuretic	386 (86.9)	401 (90.9)	406 (90.6)	0.097
ACE inhibitors or ARBs	373 (84.0)	367 (83.2)	353 (78.8)	0.092
Calcium channel blocker	154 (34.7)	173 (39.2)	211 (47.1)^*^	0.001
Hypoglycemic agent	136 (30.6)	141 (32.0)	211 (47.1)^‡^	<0.001
Other CVD medication	406 (91.4)	402 (91.2)	412 (92.0)	0.91
**Mean (±SD) of characteristic**				
Age, year	70.4 ± 9.6	71.4 ± 9.8	70.8 ± 9.4	0.30
Body mass index, kg/m^2^	32.9 ± 7.7	32.6 ± 7.1	33.4 ± 8.1	0.28
Waist circumference, cm	107.8 ± 17.4	108.2 ± 17.8	109.7 ± 18.3	0.28
Systolic pressure, mmHg	125.9 ± 13.6	127.2 ± 15.1	130.7 ± 16.2^‡^	<0.001
Diastolic pressure, mmHg	73.4 ± 11.1	72.7 ± 11.4	73.4 ± 12.2	0.63
Heart rate, beats/min	68.3 ± 11.0	68.5 ± 11.0	69.7 ± 10.9	0.14
Serum creatinine, mg/dL	1.10 ± 0.28	1.15 ± 0.31^*^	1.21 ± 0.37^†^	<0.001
eGFR, mL/min/1.73 m^2^	68.0 ± 19.8	65.0 ± 20.3^*^	63.4 ± 20.8	0.003
**Geometric mean (IQR)**				
ACR, mg/g	6.32 (5.00–9.00)	27.6 (19.0–38.0)^‡^	270.3 (113.5–562.0)^‡^	<0.001

*ACE, indicates angiotensin-converting enzyme; ARB, angiotensin receptor blockers; ACR, urine microalbumin/creatinine ratio; CVD, cardiovascular disease; eGFR, estimated glomerular filtration rate; IQR, interquartile range. p-values denote the significance of the difference in prevalence (chi-squared test) or means (ANOVA) across tertile of the distribution of serum creatinine. Significance of the difference with the adjacent lower third*:

* *p ≤ 0.05*;

† *p ≤ 0.01*;

‡ *p ≤ 0.001*.

Women compared with men had smaller (*p* < 0.001) LV end-diastolic and end-systolic volumes, septal and posterior wall thickness, and LV mass index, but higher (*p* ≤ 0.005) ejection fraction and longitudinal strain (Supplementary Table 1).

### Echocardiographic Traits Associated With ACR

In unadjusted analyses (Table 2), the association sizes in relation to each doubling of urine ACR were +0.015 cm/+0.015 cm (*p* < 0.001) for septal and posterior wall thickness, +2.43 mg/m^2^ (*p* < 0.001) for LV mass index, 0.005 (*p* = 0.012) for relative wall thickness, 0.32 (*p* = 0.047) for E/e' and 0.65 mL/m^2^ (*p* = 0.020) for left atrial volume index (Table 2). With adjustments applied for potential confounders, the associations of septal and posterior wall thickness, LV mass index, and left atrial volume index with ACR remained significant (*p* ≤ 0.003; Table 2). The septal and posterior wall thickness, LV mass index, and left atrial volume index were positively associated with urine ACR in men (*p* ≤ 0.012; Table 2), whereas only posterior wall thickness and LV mass index remained significant (*p* ≤ 0.033; Table 2) in women. The multivariable-adjusted analyses additionally accounted for serum creatine produced consistent results (Table 2).

**Table 2:** Baseline cardiac structure and function in relation to the urine microalbumin/creatinine ratio.

**Characteristics**	**Unadjusted**		**Adjusted**		**Fully adjusted**	
	**Estimates (95% CI)**	***p*-value**	**Estimates (95% CI)**	***p*-value**	**Estimates (95% CI)**	***p*-value**
**All**						
Septal wall thickness, cm	0.015 (0.006–0.023)	<0.001	0.015 (0.006–0.023)	<0.001	0.015 (0.007–0.023)	<0.001
Posterior wall thickness, cm	0.015 (0.006–0.022)	<0.001	0.014 (0.006–0.021)	<0.001	0.014 (0.006–0.021)	<0.001
LV mass index, mg/m^2^	2.43 (1.23–3.62)	<0.001	2.51 (1.30–3.72)	<0.001	2.58 (1.37–3.79)	<0.001
Relative wall thickness	0.005 (0.001–0.009)	0.012	0.004 (-0.0004–0.008)	0.078	0.004 (−0.0004–0.008)	0.073
E/A ratio	−0.026 (−0.063–0.011)	0.16	−0.021 (−0.060–0.018)	0.29	−0.018 (−0.057–0.021)	0.36
TDI e', cm/s	−0.10 (−0.23–0.044)	0.18	−0.061 (−0.21–0.087)	0.42	−0.062 (−0.21–0.086)	0.41
E/e' (septal)	0.32 (0.005–0.64)	0.047	0.20 (−0.13–0.54)	0.24	0.20 (−0.14–0.51)	0.25
Left atrial volume index, mL/m^2^	0.65 (0.10–1.19)	0.020	0.86 (0.29–1.42)	0.003	0.90 (0.33–1.46)	0.002
Ejection fraction, %	0.085 (−0.22–0.39)	0.58	0.051 (−0.25–0.36)	0.85	0.062 (−0.24–0.37)	0.69
TDI longitudinal strain, %	−0.13 (−0.32–0.060)	0.18	−0.12 (−0.33–0.072)	0.22	−0.12 (−0.31–0.073)	0.22
**Women**						
Septal wall thickness, cm	0.012 (0.001–0.024)	0.037	0.010 (−0.002–0.022)	0.11	0.010 (−0.002–0.022)	0.10
Posterior wall thickness, cm	0.013 (0.002–0.024)	0.018	0.012 (0.001–0.024)	0.033	0.012 (0.001–0.024)	0.033
LV mass index, mg/m^2^	2.56 (0.88–4.24)	0.003	2.43 (0.59–4.27)	0.010	2.45 (0.61–4.29)	0.009
Relative wall thickness	0.004 (−0.001–0.010)	0.13	0.003 (−0.003–0.009)	0.31	0.003 (−0.003–0.009)	0.31
Left atrial volume index, mL/m^2^	0.27 (−0.44–0.97)	0.46	0.36 (−0.41–1.12)	0.36	0.36 (−0.40–1.13)	0.35
**Men**						
Septal wall thickness, cm	0.018 (0.006–0.029)	0.002	0.018 (0.007–0.029)	0.002	0.018 (0.007–0.030)	0.002
Posterior wall thickness, cm	0.016 (0.005–0.027)	0.004	0.014 (0.003–0.025)	0.012	0.014 (0.003–0.025)	0.012
LV mass index, mg/m^2^	2.36 (0.74–3.98)	0.004	2.46 (0.78–4.14)	0.004	2.55 (0.85–4.26)	0.004
Relative wall thickness	0.006 (0.0003–0.012)	0.040	0.004 (−0.002–0.010)	0.16	0.004 (−0.002–0.011)	0.16
Left atrial volume index, mL/m^2^	0.98 (0.17–1.78)	0.018	1.34 (0.50–2.19)	0.002	1.44 (0.59–2.30)	0.001

*CI, indicates confidence interval; LV, left ventricular; TDI, tissue doppler imaging. Association sizes (95% CI) express the difference in indexes of cardiac structure and function associated with a doubling of urine microalbumin/creatinine ratio. Adjusted models accounted for randomly assigned treatment (spironolactone vs. placebo), sex, age, ethnicity, body mass index, systolic blood pressure, heart rate, current smoking, use of medications (diuretics, β blockers, angiotensin converting enzyme inhibitors, angiotensin receptor blockers, calcium-channel blockers, lipid-lowering drugs, aspirin, other cardiovascular medications, and hypoglycemic agents), and prevalence of diabetes mellitus and dyslipidemia. Fully adjusted models additionally adjusted for serum creatinine*.

### Risks Associated With ACR

In unadjusted models (Table 3), urine ACR predicted primary endpoint (hazard ratio [HR] for doubling increment, 1.15; *p* < 0.001), all-cause mortality (HR, 1.08; *p* = 0.003), cardiovascular mortality (HR, 1.10; *p* = 0.002), HF hospitalization (HR, 1.18; *p* < 0.001), any hospitalization (HR, 1.06; *p* < 0.001), and incidence of myocardial infarction (HR, 1.13; *p* = 0.009). In multivariable-adjusted models (Table 3), urine ACR remained predictive for those adverse health outcomes with hazard ratios ranging from 1.03 to 1.16 (*p* ≤ 0.032). There was no association between urine ACR and stroke in all models (*p* ≥ 0.062). Sensitivity analyses of various adverse outcomes in relation to urine ACR in placebo group produced confirmatory results with the exception for stroke (*p* ≤ 0.036; Table 3). In Table 3, multivariable-adjusted models additionally accounted for serum creatinine produced confirmatory results.

**Table 3:** Adverse outcomes in relation to the urine microalbumin/creatinine ratio.

**Characteristics**	**Unadjusted models**		**Adjusted models**		**Fully adjusted models**	
	**HRs (95% CI)**	***p*-value**	**HRs (95% CI)**	***p*-value**	**HRs (95% CI)**	***p*-value**
**All (*****n*** **=** **1,334)**
Primary end point (*n* = 345)	1.15 (1.11–1.20)	<0.001	1.13 (1.09–1.18)	<0.001	1.12 (1.07–1.17)	<0.001
Death (*n* = 252)	1.08 (1.03–1.13)	0.003	1.08 (1.03–1.14)	0.002	1.07 (1.01–1.12)	0.012
Cardiovascular death (*n* = 152)	1.10 (1.03–1.17)	0.002	1.10 (1.03–1.17)	0.005	1.08 (1.01–1.15)	0.023
HF hospitalization (*n* = 259)	1.18 (1.13–1.24)	<0.001	1.16 (1.10–1.22)	<0.001	1.14 (1.09–1.20)	<0.001
Any hospitalization (*n* = 709)	1.06 (1.03–1.09)	<0.001	1.03 (1.003–1.07)	0.032	1.03 (0.99–1.06)	0.10
Myocardial infarction (*n* = 60)	1.13 (1.03–1.25)	0.009	1.13 (1.02–1.26)	0.016	1.13 (1.02–1.25)	0.020
Stroke (*n* = 52)	1.10 (0.99–1.22)	0.062	1.09 (0.98–1.22)	0.13	1.08 (0.97–1.21)	0.17
**Placebo (*****n*** **=** **669)**
Primary end point (*n* = 185)	1.16 (1.10–1.22)	<0.001	1.15 (1.08–1.21)	<0.001	1.13 (1.07–1.20)	<0.001
Death (*n* = 137)	1.08 (1.01–1.15)	0.021	1.09 (1.02–1.17)	0.011	1.08 (1.01–1.16)	0.029
Cardiovascular death (*n* = 88)	1.11 (1.03–1.20)	0.007	1.13 (1.04–1.24)	0.004	1.12 (1.03–1.22)	0.010
HF hospitalization (*n* = 141)	1.18 (1.12–1.26)	<0.001	1.16 (1.09–1.24)	<0.001	1.14 (1.07–1.22)	<0.001
Any hospitalization (*n* = 359)	1.08 (1.04–1.12)	<0.001	1.05 (1.01–1.10)	0.017	1.04 (0.999–1.09)	0.052
Myocardial infarction (*n* = 29)	1.14 (0.998–1.30)	0.054	1.17 (1.004–1.36)	0.044	1.16 (0.996–1.35)	0.056
Stroke (*n* = 28)	1.15 (1.01–1.32)	0.036	1.10 (1.02–1.39)	0.028	1.17 (1.003–1.38)	0.047

*CI, indicates confidence interval; HF, heart failure; HR, hazard ratio (95% CI) express the risk of adverse outcomes associated with a doubling of urine microalbumin/creatinine ratio. Adjusted models accounted for randomly assigned treatment (spironolactone vs. placebo), sex, age, ethnicity, body mass index, systolic blood pressure, heart rate, current smoking, use of medications (diuretics, β-blockers, angiotensin converting enzyme inhibitors, angiotensin receptor blockers, calcium-channel blockers, lipid-lowering drugs, aspirin, other cardiovascular medications, and hypoglycemic agents), and prevalence of diabetes mellitus and dyslipidemia. Fully adjusted models additionally adjusted for serum creatinine*.

Table 4 shows the sex-specific risks in relation to urine ACR. In women, per doubling of urine ACR predicted primary endpoint (HR, 1.13), all-cause mortality (HR, 1.12), cardiovascular mortality (HR, 1.16), and HF hospitalization (HR, 1.12) in unadjusted models (Table 4; *p* ≤ 0.002). The corresponding multivariable-adjusted hazard ratios remained significant with the exception for HF hospitalization (*p* = 0.082; Table 4). In men, urine ACR predicted primary endpoint, HF hospitalization, any hospitalization, and incidence of myocardial infarction. The adjusted hazard ratios were 1.17, 1.25, 1.08, and 1.19 in Table 4 (*p* ≤ 0.017), respectively. The multivariable-adjusted analyses additionally accounted for serum creatine produced consistent results (Table 4). The interactions of sex with ACR were significant for hospitalization for HF and any hospitalization (*p* ≤ 0.034).

**Table 4:** Adverse outcomes in relation to the urine microalbumin/creatinine ratio by sex.

**Characteristics**	**Unadjusted models**		**Adjusted models**		**Fully adjusted models**	
	**HRs (95% CI)**	***p*-value**	**HRs (95% CI)**	***p*-value**	**HRs (95% CI)**	***p*-value**
**Women (*****n*** **=** **604)**
Primary end point (*n* = 150)	1.13 (1.07–1.19)	<0.001	1.09 (1.02–1.16)	0.010	1.08 (1.01–1.15)	0.020
Death (*n* = 100)	1.12 (1.05–1.20)	0.002	1.12 (1.03–1.21)	0.006	1.11 (1.03–1.20)	0.006
Cardiovascular death (*n* = 59)	1.16 (1.06–1.27)	0.001	1.17 (1.06–1.30)	0.002	1.17 (1.06–1.30)	0.002
HF hospitalization (*n* = 118)	1.12 (1.05–1.19)	0.001	1.07 (0.99–1.14)	0.082	1.06 (0.98–1.13)	0.14
Any hospitalization (*n* = 331)	1.03 (0.99–1.07)	0.16	0.99 (0.95–1.04)	0.81	0.99 (0.95–1.04)	0.68
Myocardial infarction (*n* = 29)	1.09 (0.95–1.25)	0.21	1.07 (0.92–1.25)	0.38	1.07 (0.92–1.24)	0.40
Stroke (*n* = 23)	1.05 (0.90–1.23)	0.54	1.05 (0.89–1.25)	0.55	1.05 (0.88–1.24)	0.61
**Men (*****n*** **=** **730)**
Primary end point (*n* = 195)	1.17 (1.11–1.24)	<0.001	1.17 (1.11–1.24)	<0.001	1.15 (1.08–1.22)	<0.001
Death (*n* = 152)	1.04 (0.98–1.11)	0.24	1.04 (0.98–1.12)	0.14	1.03 (0.96–1.10)	0.42
Cardiovascular death (*n* = 93)	1.05 (0.97–1.14)	0.22	1.05 (0.97–1.15)	0.24	1.02 (0.94–1.12)	0.60
HF hospitalization (*n* = 141)	1.26 (1.18–1.34)	<0.001	1.25 (1.17–1.34)	<0.001	1.23 (1.15–1.32)	<0.001
Any hospitalization (*n* = 378)	1.09 (1.05–1.13)	<0.001	1.08 (1.03–1.12)	0.001	1.06 (1.02–1.11)	0.006
Myocardial infarction (*n* = 31)	1.18 (1.04–1.36)	0.014	1.19 (1.03–1.36)	0.017	1.19 (1.03–1.38)	0.017
Stroke (*n* = 29)	1.15 (1.00–1.33)	0.051	1.15 (0.98–1.34)	0.080	1.14 (0.98–1.33)	0.11

*CI, indicates confidence interval; HF, heart failure; HR, hazard ratio (95% CI) express the risk of adverse outcomes associated with a doubling of urine microalbumin/creatinine ratio. Adjusted models accounted for randomly assigned treatment (spironolactone vs. placebo), age, ethnicity, body mass index, systolic blood pressure, heart rate, current smoking, use of medications (diuretics, β-blockers, angiotensin converting enzyme inhibitors, angiotensin receptor blockers, calcium-channel blockers, lipid-lowering drugs, aspirin, other cardiovascular medications, and hypoglycemic agents), and prevalence of diabetes mellitus and dyslipidemia. Fully adjusted models additionally adjusted for serum creatinine*.

**Figure 1:**
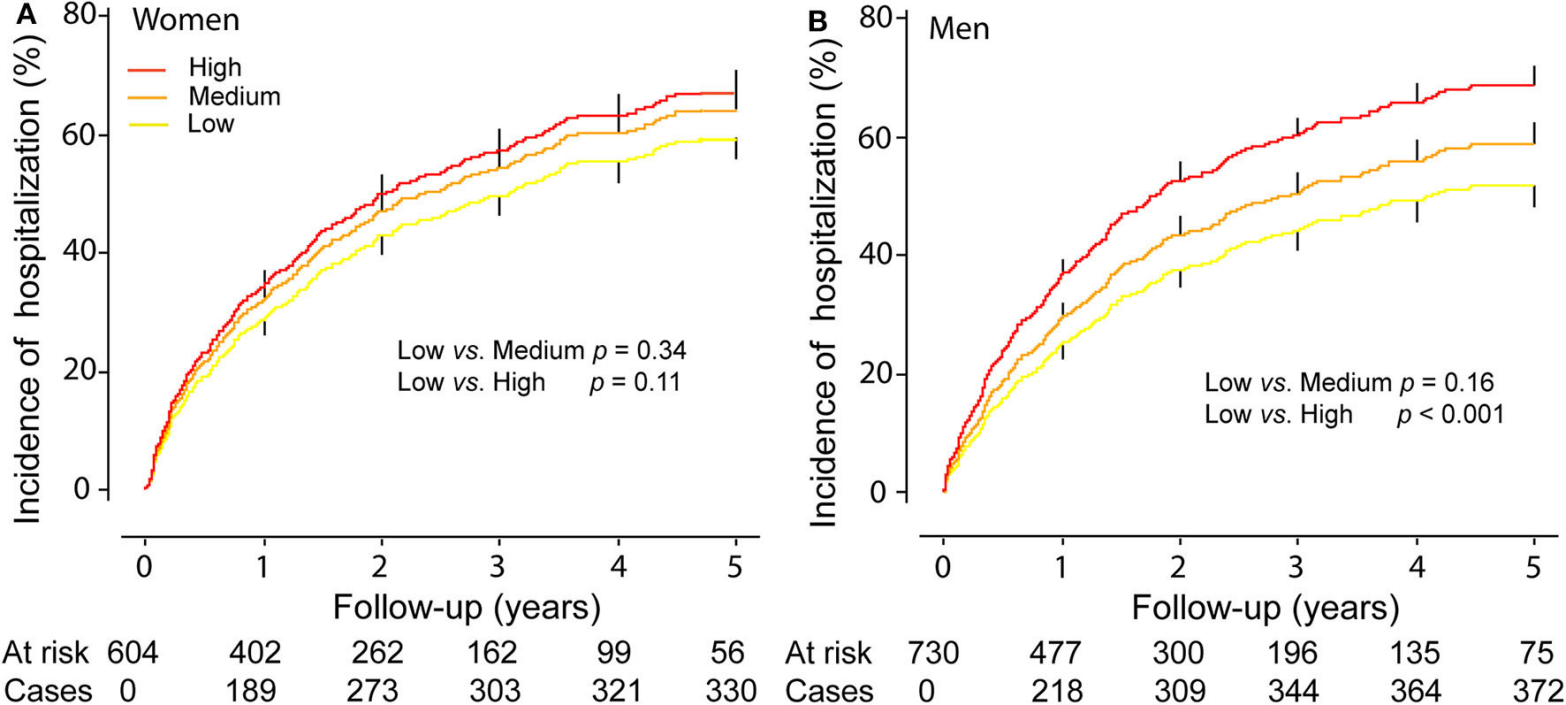
Cumulative Incidence of any hospitalization in women **(A)** and men **(B)** by tertiles of the distribution of urine microalbumin/creatinine ratio. *p*-values refer to the differences between the low and the middle and the top tertiles of urine microalbumin/creatinine ratio distribution. Vertical lines denote the standard error.

In men compared with the low tertile of ACR distribution, the incidence of any hospitalization was slightly higher in the middle tertile (*p* = 0.16) and significantly higher (*p* < 0.001) in the top tertile (Figure 1), while the incidence of any hospitalization was similar across the categories of urine ACR distribution in women (Figure 1). In women, albuminuria was associated with all-cause (1.97; CI, 1.30–3.00; *p* = 0.002) and cardiovascular (2.28; CI, 1.30–3.97; *p* = 0.004) mortality in multivariable-adjusted analyses. In men, albuminuria was important predictor of primary endpoint (HR,1.90; *p* < 0.001), HF hospitalization (HR, 2.76; *p* < 0.001), and any hospitalization (HR, 1.28; *p* = 0.025).

### Sensitivity Analysis

Sensitivity analyses of adverse health outcomes related to ACR by sex in various subgroups without diabetes mellitus (Supplementary Table 2), dyslipidemia (Supplementary Table 3), and smoking (Supplementary Table 4) generated confirmatory results.

## Discussions

The key findings can be summarized as follows: (i) in men, urine ACR was positively associated with septal and posterior wall thickness, LV mass index, and left atrial volume index, whereas in women the associations remained significant for posterior wall thickness and LV mass index; (ii) in men urine ACR predicted primary endpoint, HF hospitalization, any hospitalization, and incidence of myocardial infarction, whereas in women ACR predicted primary endpoint, all-cause mortality, and cardiovascular mortality; and (iii) the interactions of sex with ACR were significant for hospitalization for HF and any hospitalization.

Previous studies demonstrated the pre-disposition of women to HFpEF (12). The sex-specific associations between echocardiographic traits and adverse health outcomes might be partially explained by sex differences in risk factors for cardiovascular disease (19) and cardiovascular pathophysiology, including cardiac remodeling (20) and diastolic dysfunction (21). Aging, obesity, hypertension, diabetes mellitus have a different impact on cardiac and vascular structure and function, and endothelial function in women and men (20, 22, 23), indicating an important role of sex disparities in cardiovascular remodeling in patient with HF. Gori and colleagues investigated the association between sex and cardiovascular structure and function in 279 patients (mean age, 71 years; 57% women) with HFpEF from PARAMOUNT study (21). In line with our findings, they found that women had worse diastolic function (lower e' and higher E/e') and higher LV ejection fraction (21). The other mechanisms underlying women's pre-disposition to HFpEF include an activated renin-angiotensin-aldosterone system in response to low estrogen after menopause (24), calcium handling (25), and myocardial substrate metabolism (26).

Albuminuria is present in 30~50% of patients with HFpEF and confers poor prognosis (5–7). The albuminuria has been considered as a target to reduce cardiovascular outcomes in patients with HFpEF. Jackson and coworkers assessed the prevalence and prognostic value of a spot urinary ACR in 2,310 patients (mean age, 66.2 years; 33.4% women) with HF (5). In 967 patients with HFpEF, 281 (29%) had microalbuminuria and 119 (12%) had macroalbuminuria. HRs per unit ACR (100 mg/mmol) for the primary composite outcome was 1.12 (1.04–1.21) in those with HFpEF (5). In categorical analyses, HRs were 2.03 (1.45–2.85) for macroalbuminuria vs. normoalbuminuria, and 1.31 (0.99–1.74) for microalbuminuria vs. normoalbuminuria (5). The investigators of the Chronic HF Analysis and Registry in the Tohoku District 2 study demonstrated measurement of albuminuria in addition to estimated glomerular filtration rate (eGFR) is useful for risk stratification in 2,465 patients with HFpEF (6). They divided the patients into four groups: group 1 (eGFR ≥ 60, normal ACR), group 2 (eGFR ≥ 60, abnormal ACR), group 3 (eGFR <60, normal ACR), and group 4 (eGFR <60, abnormal ACR) (6). Over 2.5 years (mean) of follow-up, compared with group 1, HRs for all-cause death were 2.44 (1.47–4.05) for group 2, 1.43 (0.92–2.23) for group 3, and 2.71 (1.72–4.27) for group 4 (6).

### Study Strength and Limitations

Our current study must be interpreted within the context of its strength and potential limitations. Strengths of our study include its relatively large-sample size, long-term follow-up of this randomized clinical trial, and adjustments applied for a plenty of confounders in line with previous studies. There are some possible limitations of the study. First, we used spot measurements to estimate albuminuria. However, it is impracticable to collect 24 h urine samples in the context of a large clinical trial. Furthermore, previous studies reported good concordance between ACR estimates from spot and 24-h urine collections (27). Second, several baseline characteristics were self-reported and might have introduced recall bias in our analyses. Third, urine specimens were not available for all participants. However, participants analyzed compared with those not analyzed had similar heat rate, but were on average 3.8 years older, had a slightly higher body mass index (33.0 vs. 31.5 kg/m^2^), higher prevalence of diabetes mellitus (40.5 vs. 27.4%) and dyslipidemia (66.0 vs. 56.6%), and had a lower blood pressure (127.9 vs. 130.0 mm Hg). Finally, not all patients randomized into TOPCAT underwent echocardiography at baseline. Compared with TOPCAT participants not included in the echocardiographic study, those included were on average 1.80 years older (*p* < 0.001) and had a slightly higher body mass index (+0.71 kg/m^2^, *p* = 0.009), which, although relatively minor, may limit the generalizability of these findings. However, participants with and without baseline echocardiogram included proportionally a similar number of women, hypertensive patients, and smokers (*p* ≥ 0.093).

## Conclusion

In TOPCAT, women and men presented with different echocardiographic traits and long-term clinical outcomes in HFpEF. The interaction between ACR and sex was significant for hospitalization, and particularly, HF hospitalization. Our findings suggest that sex can be applied to better characterize patients with HFpEF regarding the association of echocardiographic traits and adverse outcomes with urine ACR. Sex-specific health promotion efforts may be warranted to improve the prevention of adverse health outcomes in both women and men. Furthermore, improving the sex-specific application of evidence-based treatments in patients with HFpEF may help reduce the observed sex disparities in various adverse health outcomes (28, 29).

## Data Availability Statement

The datasets presented in this article are not readily available because the requests to access the dataset should be sent to the NHLBI. Requests to access the datasets should be directed to https://biolincc.nhlbi.nih.gov/.

## Ethics Statement

The current analysis was approved by the ethics committee of the First Affiliated Hospital, Sun Yat-sen University, Guangzhou, China. TOPCAT complied with the Declaration of Helsinki and received ethical clearance. All patients signed informed consent prior to randomization.

## Author Contributions

JS, YD, and CL: conceptualization. F-FW, WL, and XH: formal analysis. RX, YW, YZ, MO-A, and WZ: methodology. JH, JS, YD, and CL: supervision and validation. F-FW, XH, and ZW: writing and revision. All authors interpreted the results, commented on successive versions of the manuscript, and approved the final version.

## Conflict of Interest

The authors declare that the research was conducted in the absence of any commercial or financial relationships that could be construed as a potential conflict of interest.

## Abbreviations

ACR: urine albumin/creatine ratio
CKD: chronic kidney disease
eGFR: estimated glomerular filtration rate
HF: heart failure
HFpEF: heart failure with preserved ejection fraction.
