# Supplementary material for: Sex-Specific Associations of Risks and Cardiac Structure and Function With Microalbumin/Creatinine Ratio in Diastolic Heart Failure
Source: Front Cardiovasc Med. 2020 Oct 7;7:579400. doi: 10.3389/fcvm.2020.579400 (PMC7577227; doi:10.3389/fcvm.2020.579400)
Supplement: Supplementary file 1 [file Data_Sheet_1.doc]

***Front Cardiovasc Med***

**Data Supplement**

*Sex-Specific Associations of Risks and Cardiac Structure and Function with Microalbumin/Creatinine Ratio in Diastolic Heart Failure*

Fang-Fei Wei1,2, Ruicong Xue1,2, Yuzhong Wu1,2, Weihao Liang1,2, Xin He1,2, Yuanyuan Zhou1,2, Marvin Owusu-Agyeman1,2, Zexuan Wu1,2, Wengen Zhu1,2, Jiangui He1,2, Jan A. Staessen3,4,5, Yugang Dong1,2*, Chen Liu1,2*

Author Affiliations

: 1Department of Cardiology, the First Affiliated Hospital of Sun Yat-Sen University, Guangzhou, Guangdong, China; 2NHC Key Laboratory of Assisted Circulation, Sun Yat-Sen University, Guangzhou, China; 3Studies Coordinating Centre, Research Unit Hypertension and Cardiovascular Epidemiology, Department of Cardiovascular Sciences, University of Leuven, Leuven, Belgium; 4NPA Alliance for the Promotion of Preventive Medicine, Mechelen, Belgium; 5National Guangdong Joint Engineering Laboratory for Diagnosis and Treatment of Vascular Disease, Guangzhou, China.

**^*^**Authors are equally responsible for correspondence:
Prof Chen Liu, Department of Cardiology, the First Affiliated Hospital of Sun Yat-Sen University; Email: [liuch75@mail.sysu.edu.cn](mailto:liuch75@mail.sysu.edu.cn);
Prof Yugang Dong, Department of Cardiology, the First Affiliated Hospital of Sun Yat-Sen University; Email: [dongxg@mail.sysu.edu.cn](mailto:dongxg@mail.sysu.edu.cn).

**Table of Contents**

| TABLE S1. | Baseline cardiac structure and function | p3 |
| --- | --- | --- |
| TABLE S2. | Adverse outcomes in relation to the urine microalbumin/creatinine ratio in patients without diabetes mellitus by sex | p4 |
| TABLE S3. | Adverse outcomes in relation to the urine microalbumin/creatinine ratio in patients without dyslipidemia by sex | p5 |
| TABLE S4. | Adverse outcomes in relation to the urine microalbumin/creatinine ratio in patients without smoking by sex | P6 |

**TABLE S1**

Baseline cardiac structure and function

| **Characteristics** | **Women** | | **Men** | | **All** | |
| --- | --- | --- | --- | --- | --- | --- |
|  | ***n*** | **Mean±SD** | ***n*** | **Mean±SD** | ***n*** | **Mean±SD** |
| LV structure |  |  |  |  |  |  |
| LV end-diastolic volume index, mL/m^2^ | 173 | 42.4±11.7 | 239 | 52.0±14.5^‡^ | 412 | 47.9±14.2 |
| LV end-systolic volume index, mL/m^2^ | 173 | 16.5±6.2 | 239 | 21.8±9.3^‡^ | 412 | 19.6±8.5 |
| LV end-diastolic dimension, cm | 178 | 4.63±0.49 | 254 | 4.96±0.53^‡^ | 432 | 4.82±0.54 |
| LV end-systolic dimension, cm | 178 | 3.20±0.41 | 254 | 3.50±0.46^‡^ | 432 | 3.38±0.46 |
| Septal wall thickness, cm | 178 | 1.13±0.20 | 254 | 1.27±0.21^‡^ | 432 | 1.21±0.21 |
| Posterior wall thickness, cm | 178 | 1.10±0.18 | 253 | 1.22±0.20^‡^ | 431 | 1.17±0.20 |
| LV mass index, mg/m^2^ | 178 | 101.5±28.9 | 253 | 115.6±29.8^‡^ | 431 | 109.8±30.2 |
| Relative wall thickness | 178 | 0.48±0.10 | 253 | 0.50±0.11 | 431 | 0.49±0.10 |
| LV diastolic function |  |  |  |  |  |  |
| E/A ratio | 115 | 1.24±0.61 | 130 | 1.47±0.77* | 245 | 1.36±0.71 |
| TDI e’ (lateral), cm/s | 99 | 7.96±3.6 | 126 | 8.86±3.2* | 225 | 8.46±3.4 |
| TDI e’ (septal), cm/s | 101 | 5.98±2.2 | 151 | 6.56±2.2* | 252 | 6.33±2.2 |
| E/e’(lateral) | 98 | 13.3±7.0 | 123 | 12.2±6.3 | 221 | 12.6±6.6 |
| E/e’(septal) | 100 | 16.7±6.5 | 147 | 16.0±6.9 | 247 | 16.3±6.7 |
| Left atrial volume index, mL/m^2^ | 169 | 29.6±11.3 | 232 | 31.0±14.3 | 401 | 30.4±13.1 |
| LV systolic function |  |  |  |  |  |  |
| Ejection fraction, % | 184 | 60.9±7.2 | 262 | 58.8±7.8^†^ | 446 | 59.7±7.6 |
| TDI longitudinal strain, % | 108 | 16.3±3.2 | 112 | 15.0±3.4^†^ | 220 | 15.7±3.3 |

LV indicates left ventricular; TDI, tissue Doppler imaging. Longitudinal strain is a negative value, but for ease of interpretation the absolute value was reported. Significance of the sex difference: * *^p^* ^≤ 0.05; †^ *^p^* ^≤ 0.01; ‡^ *^p^* ^≤ 0.001^.

**TABLE S2**

Adverse outcomes in relation to the urine microalbumin/creatinine ratio in patients without diabetes mellitus by sex

| **Characteristics** | **Unadjusted models** | | **Fully adjusted models** | |
| --- | --- | --- | --- | --- |
|  | **HRs (95% CI)** | ***p*-value** | **HRs (95% CI)** | ***p*-value** |
| Women (*n*=377) |  |  |  |  |
| Primary end point | 1.14 (1.04-1.25) | 0.007 | 1.12 (1.01-1.23) | 0.032 |
| Death | 1.18 (1.06-1.32) | 0.002 | 1.13 (1.01-1.27) | 0.039 |
| Cardiovascular death | 1.14 (0.99-1.31) | 0.060 | 1.12 (0.96-1.31) | 0.15 |
| HF hospitalization | 1.13 (1.01-1.26) | 0.030 | 1.11 (0.98-1.25) | 0.086 |
| Any hospitalization | 1.04 (0.98-1.11) | 0.18 | 1.02 (0.96-1.09) | 0.54 |
| Men (*n*=417) |  |  |  |  |
| Primary end point | 1.15 (1.06-1.26) | 0.001 | 1.14 (1.05-1.25) | 0.002 |
| Death | 1.05 (0.96-1.15) | 0.28 | 1.04 (0.94-1.14) | 0.48 |
| Cardiovascular death | 1.08 (0.96-1.21) | 0.20 | 1.07 (0.95-1.21) | 0.24 |
| HF hospitalization | 1.27 (1.15-1.40) | <0.001 | 1.25 (1.12-1.39) | <0.001 |
| Any hospitalization | 1.08 (1.01-1.14) | 0.017 | 1.07 (1.01-1.14) | 0.028 |

CI indicates confidence interval; HF, heart failure; HR, hazard ratio. Hazard ratios (95% CI) express the risk of adverse outcomes associated with a doubling of urine microalbumin/creatinine ratio. Fully adjusted models accounted for randomly assigned treatment (spironolactone versus placebo), age, ethnicity, body mass index, systolic blood pressure, heart rate, serum creatinine, current smoking, use of medications (diuretics, β‑blockers, angiotensin converting enzyme inhibitors, angiotensin receptor blockers, calcium-channel blockers, lipid-lowering drugs, aspirin, other cardiovascular medications, and hypoglycemic agents), and prevalence of dyslipidemia.

**TABLE S3**

Adverse outcomes in relation to the urine microalbumin/creatinine ratio in patients without dyslipidemia by sex

| **Characteristics** | **Unadjusted models** | | **Fully adjusted models** | |
| --- | --- | --- | --- | --- |
|  | **HRs (95% CI)** | ***p*-value** | **HRs (95% CI)** | ***p*-value** |
| Women (*n*=220) |  |  |  |  |
| Primary end point | 1.14 (1.01-1.29) | 0.035 | 1.11 (0.96-1.27) | 0.15 |
| Death | 1.20 (1.07-1.35) | 0.002 | 1.10 (0.95-1.26) | 0.20 |
| Cardiovascular death | 1.26 (1.08-1.46) | 0.003 | 1.26 (1.02-1.56) | 0.032 |
| HF hospitalization | 1.06 (0.91-1.23) | 0.48 | 1.03 (0.87-1.22) | 0.72 |
| Any hospitalization | 1.03 (0.95-1.12) | 0.47 | 1.02 (0.93-1.11) | 0.70 |
| Men (*n*=234) |  |  |  |  |
| Primary end point | 1.30 (1.17-1.44) | <0.001 | 1.31 (1.17-1.48) | <0.001 |
| Death | 1.13 (0.999-1.27) | 0.051 | 1.18 (1.02-1.36) | 0.024 |
| Cardiovascular death | 1.12 (0.96-1.30) | 0.15 | 1.16 (0.98-1.38) | 0.090 |
| HF hospitalization | 1.49 (1.30-1.70) | <0.001 | 1.53 (1.30-1.80) | <0.001 |
| Any hospitalization | 1.11 (1.03-1.21) | 0.009 | 1.12 (1.02-1.22) | 0.016 |

CI indicates confidence interval; HF, heart failure; HR, hazard ratio. Hazard ratios (95% CI) express the risk of adverse outcomes associated with a doubling of urine microalbumin/creatinine ratio. Fully adjusted models accounted for randomly assigned treatment (spironolactone versus placebo), age, ethnicity, body mass index, systolic blood pressure, heart rate, serum creatinine, current smoking, use of medications (diuretics, β‑blockers, angiotensin converting enzyme inhibitors, angiotensin receptor blockers, calcium-channel blockers, lipid-lowering drugs, aspirin, other cardiovascular medications, and hypoglycemic agents), and prevalence of diabetes mellitus.

**TABLE S4**

Adverse outcomes in relation to the urine microalbumin/creatinine ratio in patients without smoking by sex

| **Characteristics** | **Unadjusted models** | | **Fully adjusted models** | |
| --- | --- | --- | --- | --- |
|  | **HRs (95% CI)** | ***p-*value** | **HRs (95% CI)** | ***p*-value** |
| Women (*n*=581) |  |  |  |  |
| Primary end point | 1.13 (1.06-1.20) | <0.001 | 1.08 (1.01-1.15) | 0.021 |
| Death | 1.12 (1.04-1.21) | 0.002 | 1.12 (1.03-1.22) | 0.006 |
| Cardiovascular death | 1.16 (1.06-1.28) | 0.002 | 1.19 (1.07-1.33) | 0.001 |
| HF hospitalization | 1.12 (1.04-1.19) | 0.001 | 1.05 (0.98-1.13) | 0.16 |
| Any hospitalization | 1.03 (0.99-1.07) | 0.16 | 0.99 (0.95-1.04) | 0.71 |
| Men (*n*=649) |  |  |  |  |
| Primary end point | 1.15 (1.08-1.22) | <0.001 | 1.14 (1.06-1.21) | <0.001 |
| Death | 1.02 (0.95-1.09) | 0.59 | 1.02 (0.95-1.10) | 0.62 |
| Cardiovascular death | 1.04 (0.95-1.14) | 0.36 | 1.02 (0.93-1.12) | 0.66 |
| HF hospitalization | 1.23 (1.15-1.31) | <0.001 | 1.21 (1.13-1.31) | <0.001 |
| Any hospitalization | 1.07 (1.03-1.12) | 0.002 | 1.06 (1.01-1.11) | 0.023 |

CI indicates confidence interval; HF, heart failure; HR, hazard ratio. Hazard ratios (95% CI) express the risk of adverse outcomes associated with a doubling of urine microalbumin/creatinine ratio. Fully adjusted models accounted for randomly assigned treatment (spironolactone versus placebo), age, ethnicity, body mass index, systolic blood pressure, heart rate, serum creatinine, use of medications (diuretics, β‑blockers, angiotensin converting enzyme inhibitors, angiotensin receptor blockers, calcium-channel blockers, lipid-lowering drugs, aspirin, other cardiovascular medications, and hypoglycemic agents), and prevalence of diabetes mellitus and dyslipidemia.
